# Manta Matcher: automated photographic identification of manta rays using keypoint features

**DOI:** 10.1002/ece3.587

**Published:** 2013-05-22

**Authors:** Christopher Town, Andrea Marshall, Nutthaporn Sethasathien

**Affiliations:** 1 Computer Laboratory, University of Cambridge15 JJ Thomson Avenue, Cambridge, CB3 0FD, UK; 2 Manta Ray and Whale Shark Research Centre, Marine Megafauna FoundationTofo Beach, Inhambane, Mozambique; 3 School of Informatics, University of Edinburgh11 Crichton Street, Edinburgh, EH8 9LE, UK

**Keywords:** Computer-aided pattern recognition, conservation biology, marine and fisheries management, photographic identification

## Abstract

For species which bear unique markings, such as natural spot patterning, field work has become increasingly more reliant on visual identification to recognize and catalog particular specimens or to monitor individuals within populations. While many species of interest exhibit characteristic markings that in principle allow individuals to be identified from photographs, scientists are often faced with the task of matching observations against databases of hundreds or thousands of images. We present a novel technique for automated identification of manta rays (*Manta alfredi* and *Manta birostris*) by means of a pattern-matching algorithm applied to images of their ventral surface area. Automated visual identification has recently been developed for several species. However, such methods are typically limited to animals that can be photographed above water, or whose markings exhibit high contrast and appear in regular constellations. While manta rays bear natural patterning across their ventral surface, these patterns vary greatly in their size, shape, contrast, and spatial distribution. Our method is the first to have proven successful at achieving high matching accuracies on a large corpus of manta ray images taken under challenging underwater conditions. Our method is based on automated extraction and matching of keypoint features using the Scale-Invariant Feature Transform (SIFT) algorithm. In order to cope with the considerable variation in quality of underwater photographs, we also incorporate preprocessing and image enhancement steps. Furthermore, we use a novel pattern-matching approach that results in better accuracy than the standard SIFT approach and other alternative methods. We present quantitative evaluation results on a data set of 720 images of manta rays taken under widely different conditions. We describe a novel automated pattern representation and matching method that can be used to identify individual manta rays from photographs. The method has been incorporated into a website (mantamatcher.org) which will serve as a global resource for ecological and conservation research. It will allow researchers to manage and track sightings data to establish important life-history parameters as well as determine other ecological data such as abundance, range, movement patterns, and structure of manta ray populations across the world.

## Introduction

The identification of individuals of a particular species is a vital requirement for many aspects of ecological research and conservation (Couturier et al. 2012). Tagging of animals is often infeasible on a large scale due to costs, effort required for tag application, low probability of tag return, and the risk of disturbing local study populations (Reisser et al. 2008). An increasingly popular alternative is visual identification, which has become an established technique employed to study animals exhibiting natural markings that are sufficiently stable over time to allow individuals to be distinguished (Hammond et al. 1990; Marshall and Pierce 2012).

However, the complexity and subtleties of many such markings, combined with the desire for ever larger study populations, mean that there is a growing need for automated visual identification techniques to aid researchers and minimize the increasing effort and chance of errors (Morrison et al. 2011; Chesser 2012) arising from manual pairwise comparisons across large image corpora. Unfortunately, whereas good accuracies have been achieved in automated photographic identification systems for many species observed on land or at the surface of the sea (Burghardt and Campbell 2007; Mortensen et al. 2007; Buonantony 2008; Gamble et al. 2008; Kniest et al. 2010; de Zeeuw et al. 2010; Hoque et al. 2011; Morrison et al. 2011; Bolger et al. 2012; Thornycroft and Booth 2012), recognition of animals underwater poses significant additional challenges. The problems of visual identification are therefore exacerbated in the case of purely aquatic animals such as elasmobranchs (Marshall and Pierce 2012), as photographs of such animals are taken under challenging underwater imaging conditions exhibiting highly variable lighting and visibility, as well as variation in pose angle, distance from the subject, flexion of the ray's body, and occlusions (part of the animal being obscured by other fish).

Various computer-aided tools have been devised to help marine scientists and conservationists with visual identification. Generally, they take a candidate image of an animal as input and produce a ranked list of possible matching individuals. A number of such systems have been shown to work well for species which frequently exhibit fairly regular or high-contrast spot patterns such as whale sharks *Rhincodon typus* (Holmberg et al. 2008, 2009; Brooks et al. 2010), ragged-tooth sharks *Carcharias taurus* (Tienhoven et al. 2007), and coelacanths *Latimeria spp*. (Thornycroft and Booth 2012).

One of the most popular tools is the I^3^S system^1^ (Speed et al. 2007; Tienhoven et al. 2007). It requires the user to perform many initial manipulations in the preprocessing stages to select reference points and identify the most distinctive spots.^2^ After that, the pattern-matching method is done by using a 2D affine transformation on the reference points to transform two images into a commonly defined plane and then compute distances between user-selected spots on two images in this plane to calculate a similarity score.

However, none of the existing tools have achieved satisfactory performance on manta ray (*Manta alfredi* and *M. birostris*) images due to the generally low contrast and high variation in size and shape exhibited by their characteristic ventral body markings, which typically consist of amorphously shaped spots and patches (Marshall and Pierce 2012). Recently an extension called I^3^S manta was proposed to deal with species such as manta rays with irregular spot patterns, but no large-scale evaluation data on its performance are currently available, and the high degree of user effort combined with unproven accuracy have hampered its adoption by scientists. Manual marker identification significantly aids automated pattern matching, but is very cumbersome and hence does not easily scale to larger data sets such as those considered in this study. For example, the application of I^3^S to coelacanth images (Thornycroft and Booth 2012) required 60 diagnostic spots to be manually defined on each of 29 images.

Furthermore, many methods assume that the region of interest (ROI) containing the characteristic spot patterns are on a near-planar or generalized cylindrical surface and only the relative position of the spots to one another is important, whereas their size is not. Unlike many bony fishes and sharks whose ROI is fairly rigid and exhibits a regular pattern, manta rays' ROI often encompasses their highly flexible wing-like pectoral fins and the pattern shows wide variation from small regular spots to frayed blotches.

Whereas tools such as I^3^S require very substantial user effort, the “mantamatcher” method described in this study is highly automated: images can be inducted in a few seconds and image matching queries are fast (a few seconds for hundreds of images on a single laptop computer). Manual image preprocessing is minimal, characteristic features are extracted automatically, and matching speed is orders of magnitude faster than comparable systems such as that proposed by (Arzoumanian et al. 2005).

Our method employs the Scale-Invariant Feature Transform (SIFT) (Lowe 2004), an algorithm to detect and describe local image features. These features are defined such that they are robust to changes in location, scale and rotation, and partially invariant to changes in illumination, noise, and minor changes in 3D camera viewpoint. Furthermore, they are also highly distinctive in the sense that a single feature can be correctly matched against a large number of features with high probability. SIFT has previously been applied in ecological research, for example, de Zeeuw et al. (2010) have designed a system based on the SIFT algorithm to assist land photo identification of leatherback sea turtles using the unique pink spots on their heads. Their preprocessing step involves manual cropping and automatic highlighting of the spots so as to reduce the effects of varying illumination, resolution, and viewing angle. The SIFT algorithm is then applied to the resulting image to get representative features that are then matched against each image in the database in turn. Similar approaches have been applied to match images of insects (Mortensen et al. 2007) and butterflies (Hao et al. 2009).

However, all these approaches require the ROI to be almost parallel to the optical plane of the camera and do not consider underwater images. The performance of standard SIFT can degrade badly in the presence of noise, occlusions, and large changes to 3D viewpoint, all of which are common in underwater images of manta rays. We therefore propose a number of modifications and enhancements of the SIFT algorithm. Moreover, we show how the accuracy and robustness of the resulting “mantamatcher” algorithm outperforms very recently proposed state-of-the-art computer vision algorithms such as Speeded Up Robust Features (SURF) (Bay et al. 2006) and ORB (Oriented FAST and Rotated BRIEF) (Rublee et al. 2011). Finally, we discuss how the mantamatcher (*MM*) method is driving development of a global collaborative resource for research into manta ray ecology.

## Materials and Methods

### Data sets

We present quantitative evaluation results on a data set of 720 images of 265 different manta rays taken under widely different conditions. The images were taken by members and associates of the Manta Ray & Whale Shark Research Centre, Marine Megafauna Foundation, Tofo Beach, Inhambane, Mozambique. Most of the images (581 photos of 214 individuals) depict reef manta rays *M. alfredi* (Marshall et al. 2011), and the remainder (139 photos of 51 individuals) depict giant manta rays *M. birostris*. Of the 214 reef mantas, 161 were visually assessed as being female and 51 as being male (there were two rays for which sex could not be determined), and of the 51 giant mantas, 38 were visually assessed as being female and 6 as being male (with the remaining 7 being indeterminable).

### Algorithm overview

Our *MM* method consists of the following main stages:

- Image preprocessing: This is a simple alignment process in which the user normalizes the 2D orientation of the ray within the image and selects a rectangular ROI containing the spot pattern.
- Image enhancement: The image is automatically enhanced through noise removal and adaptive contrast equalization.
- Feature extraction: Characteristic features of the spot pattern are automatically identified and encoded without any need for user input.
- Feature matching: Given an image of an unknown specimen and a database of manta ray sightings, the system automatically produces a ranked list of previously identified manta rays that best match it, and also provides similarity and confidence scores to allow the user to assess whether the specimen has previously been sighted.

### Image preprocessing and enhancement

**Figure 1:**
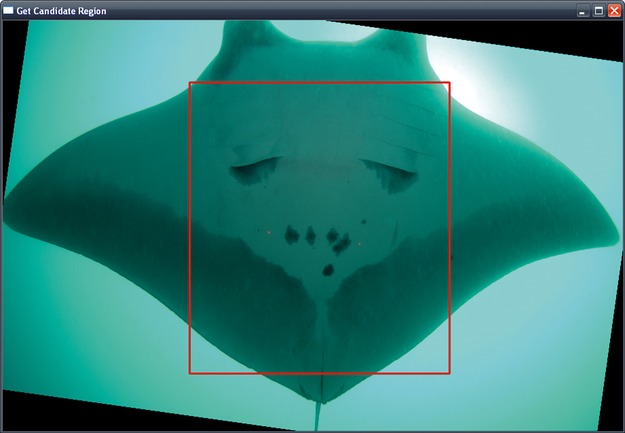
Image preprocessing: The manta ray orientation is normalized, and a candidate region encompassing the spot pattern is selected.

The preprocessing step consists of normalizing the in-plane orientation of the manta ray within the image (the user simply performs two mouse clicks to identify the orientation of the medial axis line), followed by selection of a ROI encompassing the characteristic markings on the ventral surface (Marshall et al. 2011) of the ray (this requires a further two mouse clicks to select the two opposite corners of a rectangle). Preprocessing is currently done manually, although an automated approach using image segmentation is under development. Figure 1 shows an orientation normalized manta ray image with rectangular spot pattern region.

An automated image enhancement step is then applied to the ROI image. Underwater images exhibit enormous variation in lighting depending upon factors such as depth, clarity of the water, use of flash (which often leads to backscatter caused by suspended particles), and the relative position of the sun (manta rays are frequently photographed swimming overhead with the sun behind them, causing glare and a “corona effect”). As noted in Schettini and Corchs (2010), this leads to limited visibility, low contrast, nonuniform lighting, blurring, lack of coloration, and various noise artifacts.

Given the heterogeneity of photographic conditions and equipment used in acquiring manta ray photo ID images, and the lack of usable calibration information, automated image restoration or illumination correction methods such as those proposed in Schettini and Corchs (2010) are not applicable. We therefore investigated a large number of generic and robust techniques for automated image enhancement that would be universally applicable to deal with the most commonly encountered image degradations without negatively impacting images that were already of a high quality.

Best overall results were achieved by a combination of median filtering and histogram equalization (Gonzalez and Woods 2008). Images are first converted into grayscale and then size normalized such that their maximal dimension does not exceed 800 pixels. We then calculate the standard deviation to assess noise levels and apply a 3 × 3 (for less noisy images) or 5 × 5 (for images with high noise levels) median filter to reduce noise. To improve fidelity and enhance the contrast of characteristic spot patterns, we then apply contrast-limited adaptive histogram equalization (CLAHE). The CLAHE algorithm performs local rather than global contrast adjustment, which is especially important when different parts of a manta ray in a given image exhibit widely different illumination levels, for example, due to rapid attenuation of flash lighting causing significant differences in white balance between areas of the ray that are proximate to the camera and those that are further away.

**Figure 2:**
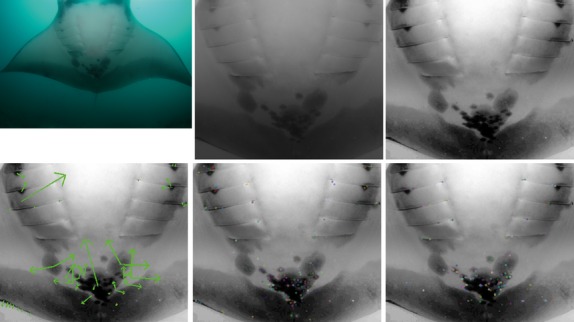
Top row, left to right: Original image; grayscale candidate region; enhanced image after noise filtering and contrast adjustment. Bottom row: Visualization of features extracted using SIFT (left), SURF (middle), and ORB (right).

An example of the effects of image enhancement is shown in Figure 2.

### Feature extraction and representation

In order to encode the characteristic information contained within the natural body markings of manta rays, we make use of the Scale-Invariant Feature Transform (SIFT) (Lowe 2004). We also implemented and evaluated two other recent feature extraction algorithms, namely SURF (Bay et al. 2006) and ORB (Rublee et al. 2011). Our implementation makes use of the OpenCV^3^ Computer Vision library (Bradski and Kaehler 2008), and we have adapted SIFT code originally written by Hess (Hess 2010).

All three algorithms detect distinctive features at keypoints in the image, and then represent those features in terms of a parametric description of the local image variation in the vicinity of the keypoints at a carefully chosen scale of analysis. The algorithms were chosen due to their ability to extract and match features in a way which is robust to changes in size and 2D rotation, and also resilient to changes in 3D viewpoint, addition of noise, and change in illumination. As we achieved best results using SIFT, and as we developed various novel improvements to the original SIFT algorithm to enhance matching performance on manta ray images, we will briefly describe some key aspects of our SIFT implementation.

To find stable features that are invariant to size, SIFT detects features using a scale-space approach. This is achieved by convolving the image with Gaussian filters *G* at different scales of analysis σ and differencing the resulting blurred images at neighboring scales to find local minima and maxima. Formally, the scale space *L* of an image is created by convolving the input image *I* with Gaussian filters *G* at different scales σ:


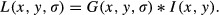


Neighboring scales (σ and *k*σ for some constant *k*) are then subtracted from each other to produce the Difference-of-Gaussian images *D*:


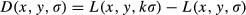


Only scale-space extrema of *D*(*x*, *y*, *σ*) that have strong contrast are chosen as keypoints. We also reject keypoints that are closely spaced along an edge as these are unstable and not useful for identification.

In order to achieve invariance to 2D orientation, a keypoint descriptor based on local gradient directions and magnitudes is used. The descriptor is invariant to image rotations as the bins of the orientation histograms are normalized relative to the dominant gradient direction in the vicinity of the keypoint. The scale of analysis, and hence the size of the local region whose features are being represented, corresponds to the scale at which the given keypoint was found to be a stable extremum (subject to constraints on local contrast and contour membership).

In terms of the characteristic patterns present on the ventral surface of manta rays, SIFT keypoints are typically localized at significant spots and other markings. Information on the shape, contrast, and dominant orientation of markings is represented by the feature descriptors.

Figure 2 illustrates image enhancement and features extraction using an example manta ray image. SIFT features are marked by arrows whose length and direction illustrate the scale and dominant orientation of the given keypoint feature (note that some keypoint features may be localized at or near the same pixel coordinates in the image, but will differ in their scale or orientation). SURF and ORB features are indicated by small circles.

### Pattern matching for automated identification in ecological databases

In order to identify a manta ray automatically from an image, it needs to be matched against all images in a database. If other images of the given individual ray are already present in the database, then the software should rank that individual highly in the list of search results. If the image represents an as-yet-unidentified individual, then the matching algorithm should return very low matching scores and indicate a low confidence of having achieved a successful match.

Matching therefore requires the software to efficiently compute all possible pairwise matches between the features representing the query image and each image in the database. Configurations of SIFT keypoints from different images can be compared via a distance metric to find correspondences between instances of objects in different poses. Most approaches to matching of SIFT features are designed for tasks such as image stitching or detection of man-made objects. In these cases it is usually straightforward to identify subsets of features representing similar or identical image structures. To achieve partial pose invariance, a candidate match can be confirmed or rejected by establishing a projective mapping of one (sub)image to another via homographies.

However, our experiments showed that this approach does not yield usable results in the case of manta ray images. The diffuse nature of their natural body markings, especially when photographed underwater, typically results in relatively low numbers of matching features, and the much greater variation in appearance caused by the factors mentioned in previous sections makes it very difficult to automatically recover the 3D pose of the ray.

We therefore developed a novel image-to-image matching method which is more akin to methods for computing similarity of visual textures as opposed to rigid transformations of geometrical or strongly patterned objects. The overall similarity score between the unknown query image *I* and another image *J* is computed as


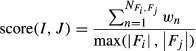


where *F*_*i*_ and *F*_*j*_ represent the sets of SIFT features for *I* and *J*, respectively. In Lowe's original algorithm (henceforth referred to as “classic SIFT”), score(*I*, *J*) is simply computed as the number of features that are deemed to match 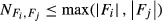 divided by the larger of the number of features in the two images. Our *MM* algorithm refines this by weighting each matched pair of features based on their significance and the strength of the match, as will be explained below. The final score is normalized based on the maximum possible value of 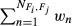 in order to ensure that scores range from 0 (worst score) to 1 (perfect match).

First, our tool considers all possible pairings of individual features from the unknown query image and all the images it is to be compared against. As each image may have hundreds of features and the database may contain thousands of images, matching feature pairs are identified efficiently using a “Best-Bin-First” (BBF) k-d-tree approximation (Beis and Lowe 1997) to the nearest neighbor Euclidean distance between feature vectors, resulting in a significant (100×) speedup.

In keeping with classic SIFT, we only consider features to be matched if their nearest neighbor to second-nearest neighbor feature distance ratio is greater than a threshold (in this case, a threshold of 0.75 was empirically chosen). This ensures that chosen features are sufficiently distinctive relative to the overall feature set.

In addition, candidate matching feature pairs are rejected if the ratio of their absolute-scale difference divided by the greater of the two scales exceeds a threshold of 0.5. This ensures that features must not differ greatly in size (i.e., by no more than one scale-space octave) to be considered to be matched. As features at a very fine scale are less likely to be significant, we ignore features with a very low scale (a scale-space value of 1.1 was empirically selected as a good cut-off) and weight the contribution (see *w*_*n*_ above) of matching keypoint pairs based on their absolute and relative scales:


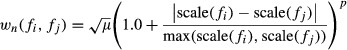


where *f*_*i*_ ∈ *F*_*i*_ and *f*_*j*_ ∈ *F*_*j*_ are the candidate features in images *I* and *J*, 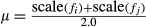 is their mean scale, and a value of *P* = 0.10 yielded best results on an evaluation set.

The crucial factor which is captured by this algorithm is that the distinctiveness (discriminability) of features is the most important factor in finding good matches, rather than just the number of similar features. Images with a fairly random distribution of features could lead to many incidentally matching features, but these may not be considered as useful matches unless they are also relatively distinctive (as determined using the nearest neighbor distance ratio), and even if they pass the test for distinctiveness, their contribution to the overall matching score is normalized based on scale and the overall number of features in both images.

We also ensure that keypoint matches are unique, that is, the same feature in one image can only be “paired” with a feature in another image once. Furthermore, the image-to-image feature comparison is computed bidirectionally, with the lowest of the two directional scores being used as the final matching score:


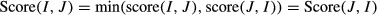


This removes bias that might otherwise result if the two images differ greatly in their feature complexity (due to the BBF approximation when computing feature distances), and also ensures that the final scores are symmetric.

In practice we are interested in matching an image *I* depicting an unknown manta ray against a set of labeled images *J* to establish whether *I* shows one of the *M* manta rays in *J* or whether it is a “new” (as-yet-unidentified) ray. Hence, *J* is effectively partitioned into subsets *J*_*m*_ for each manta ray *m* ∈ *M*, as there are likely to be multiple images of most rays (i.e., each *J*_*m*_ usually contains more than one image) in an ecological database. Our *MM* algorithm exploits this fact by first computing the pairwise (bidirectional, as above) comparisons between *I* and all the elements of each set *J*_*m*_. We then combine the resulting image-to-image scores for *J*_*m*_ by computing their mean, and use this as the overall similarity score between image *I* and manta ray *m*:


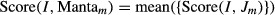


In our experiments we considered using the mean, median, maximum, and minimum as the score combination criterion, but consistently achieved best results using the mean.

**Figure 3:**
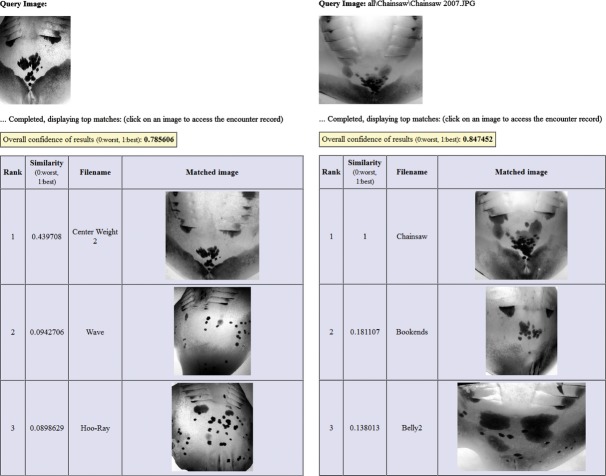
Example retrieval results (only top three candidate matches are shown).

We then sort these scores and output a ranked list of manta rays in decreasing order of how well they match the query. Consequently, the image *I* is then deemed to be best matched to the manta ray *m* with the highest similarity score, but by outputting a ranked list of results we allow the user to make the final decision as to which manta ray (if any) is the “correct” match. Figure 3 shows two examples of this: in each case, an unknown manta ray is used as the “query image” and the system displays a ranked list of the best matching manta ray images from a database (only the top three matches are shown in the figure). In order to give the user some indication of how reliable the system's rankings are likely to be, it also computes a “confidence score” based on the ratio of the scores between the first and second ranked results:


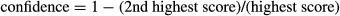


A high confidence number is an indication that the best matching image is significantly more similar to the query than any other image. If the confidence is low, then the user may need to inspect a larger number of matching results to ascertain which (if any) of them actually matches the query image.

## Results

### Quantitative and qualitative evaluation

The performance of the automated manta ray–matching methods presented in this study was evaluated using the images described in the section entitled Data sets. To assess how effective our algorithms are for automatically identifying images of manta rays, we iterate through all the images in each data set and for every such image we automatically match it (the “query image”) against all the other images in that data set. As our data sets contain at least two different images (corresponding to separate encounters) of every manta ray, we record the rank at which our algorithm matches each query image against the other images that show the same manta ray (as determined using the “mean score” criterion described above).

A perfect match would correspond to the correct manta ray being returned as the top-ranked matching result in every case. If the match is not perfect, then manual inspection of results (see Fig. 3) still makes it fairly straightforward to identify the correct manta ray as long as it appears within the first few dozen results. In that case the system is of clear value as an automated visual identification tool. Even if several top-ranked images had to be visually inspected, our system would substantially reduce the manual effort currently required by ecologists preforming exhaustive searches of entire databases (e.g., hundreds or thousands of individuals).

**Figure 4:**
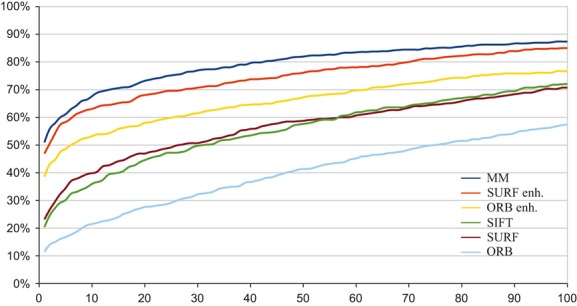
Graphs showing the cumulative distribution of successful retrieval ranks using different matching algorithms for all the images in a data sets of 720 manta ray photos. The graphs plot the cumulative proportion of correctly retrieved images against the rank at which such images are correctly matched by each algorithm (see text for details).

We therefore quantify the performance of different algorithms by analyzing and plotting the cumulative distribution of retrieval ranks for all the images in our data sets. Results on the complete data set of 720 manta ray images are shown in Figure 4. The graph contrasts the performance of six different matching algorithms:

1. *MM*: This is our new “mantamatcher” algorithm using SIFT features and the enhanced matching algorithm detailed in the previous sections.
2. *SIFT*: This method also uses SIFT features, but with default parameters and the standard SIFT matching method (but not using homographies) as described in Lowe (2004).
3. *SURF* and *ORB*: These use SURF and ORB features, respectively. Features are extracted using parameters as described in the literature (Bay et al. 2006; Rublee et al. 2011) and compared using “brute-force” matching (i.e., not using the BBF speedup used for the *MM* and SIFT methods) with the distance criterion being the Euclidean norm.
4. *SURF enh*. and *ORB enh*.: These methods are customized variants of the *SURF* and *ORB* methods described above. They make use of the same features, but performance has been enhanced by implementing a variant of our new matching algorithm to match those features (the main differences being that feature distances are computed using the Hamming distance as implemented using OpenCV and are not weighted by features scale).

Figure 4 clearly shows that the *MM* algorithm outperforms all the other methods. The default *ORB* method performs poorly, as do the default *SIFT* and *SURF* approaches. However, the *SURF enh*. and *ORB enh*. methods work relatively well, which again demonstrates the effectiveness of the new matching algorithm introduced in this study.

In order to more precisely quantify and contrast these performance differences, we also tabulate the cumulative percentages for the number of images that are correctly matched at different ranks. As Table 1 shows, *MM* perfectly matches most of the 720 images at top rank, whereas over three quarters are correctly matched within the top 25 results and over 84% within the top 72 (corresponding to 10% of the total number of images, which is sometimes used as a performance metric in related research publications). By contrast, the standard *SIFT*, *SURF*, and *ORB* methods achieve <50% accuracy in the top 25 results.

**Table 1:** Performance statistics of different matching algorithms for all the images in a data sets of 720 manta ray photos

Algorithm	Cumulative proportion of correctly matched manta ray images by retrieval rank						Cohen's D

	Top 1 (%)	Top 10 (%)	Top 25 (%)	Top 50 (%)	Top 72 (%)	Top 100 (%)	
*MM*	50.97	67.64	75.14	81.94	84.44	87.36	1.340
*SURF enh*.	46.94	63.06	69.58	76.11	80.83	85.00	0.896
*ORB enh*.	38.61	53.19	59.86	67.22	72.50	76.67	0.587
*SURF*	23.19	39.86	49.17	58.75	64.03	70.69	0.618
*SIFT*	20.42	35.97	47.22	57.64	64.72	72.08	0.727
*ORB*	11.53	21.53	29.17	41.39	49.17	57.36	0.365

Another approach to evaluation is to view visual identification as a classification problem and focus on the similarity scores rather than the ranking distribution. A good classifier needs to achieve high discriminability (decidability) between images that belong to the same class (manta ray) and those that do not, assigning high scores to the former and relatively low scores to the latter. One common statistical tool for quantifying discriminability is *Cohen's D* coefficient, which we compute from the full distribution of similarity scores for all subsets of images of the same manta ray as opposed to all the other images:


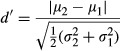


where the two distributions are characterized by means *μ*_1_ and *μ*_2_ and standard deviations *σ*_1_ and *σ*_2_. When used as a descriptive statistic to quantify effect size, a value of *d*′ < 0.2 is usually regarded as low, whereas values of *d*′ > 0.8 are considered high. Table 1 shows that our proposed *MM* method yields a very high value of *d*^′^ = 1.34.

**Figure 5:**
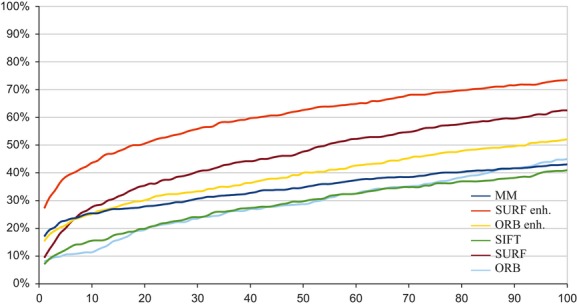
Graphs of matching accuracy using the same methodology and data set as in Figure 4, but without using any of the proposed image enhancements.

We next consider the impact of the automated image enhancement steps described above. As the graph in Figure 5 clearly shows, the automated noise removal and contrast enhancement greatly improve matching performance. Without them the performance of all of the algorithms degrades badly, although the *SURF enh*. method is somewhat less affected by this than the others.

Another factor we wish to analyze is database size and composition. In practice it may be possible to restrict the subset of the database that needs to be searched (either manually or with help of an automated system) by utilizing other factors such as the sex, species, or other physical attributes (e.g., size or bite mark scars caused by sharks) of an unknown manta ray.

**Figure 6:**
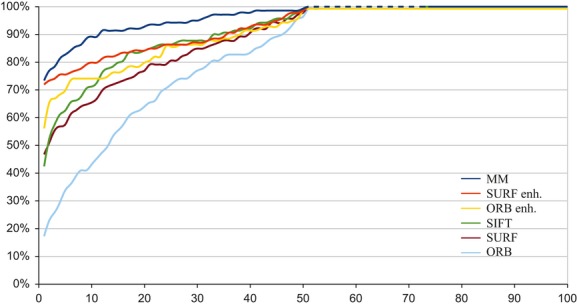
Graphs of matching accuracy on a data set of 139 photos of giant manta rays.

**Figure 7:**
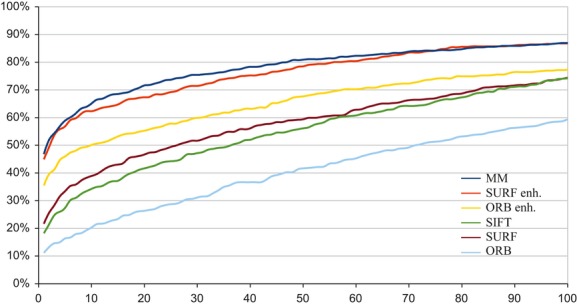
Graphs of matching accuracy on a data set of 581 photos of reef manta rays.

Figure 6 show rank-based matching performance on a subset consisting of 139 photos of giant manta rays, and Figure 7 shows performance on the remaining 581 reef manta ray images in our data set. As before, the *MM* algorithm outperforms all the other methods. As expected, performance on the smaller giant manta data set is significantly better. Surprisingly, results on the reef manta ray images are less accurate than on the full data set of 720 images. This may be due to giant manta rays having more distinctive spot patterns, or reef mantas having greater variability in markings.

We also evaluated the performance of our matching method on images of subsets consisting of only male or female reef manta rays, respectively. The sex of mature mantas can be easily assessed by visual inspection of the pelvic fin region in most cases, and ecological databases of mantas often exhibit a female sex bias. There is also some evidence for sexual segregation among manta rays (Marshall and Bennett 2010). Table 2 shows cumulative percentages for the number of images that are correctly matched at different ranks using the *MM* algorithm, and also the *Cohen's D* coefficient. It can be seen that accuracy for the set of giant mantas is substantially better than for the set of male reef mantas, even though both sets contain very similar numbers of images. Allowing for differences in data set size, there are no significant differences in accuracy on the male-only and female-only data sets: both have similar values of *d*′, and the cumulative retrieval achieved at a rank corresponding to 10% of the total number of images is also similar (about 80%).

**Table 2:** Performance statistics of the mantamatcher (*MM*) matching algorithm for different subsets of the set of 720 manta ray photos

Image subset	Cumulative proportion of correctly matched manta ray images by retrieval rank					Cohen's D

	Top 1 (%)	Top 10 (%)	Top 25 (%)	Top 50 (%)	Top 100 (%)	
Giant mantas (139 images)	73.38	89.21	94.24	99.28	100.00	1.732
Reef mantas (581 images)	46.82	65.06	73.67	80.90	86.92	1.232
Male reef mantas (119 images)	57.14	82.35	89.08	99.16	100.00	1.394
Female reef mantas (457 images)	47.26	66.30	75.49	81.40	90.59	1.191

**Figure 8:**
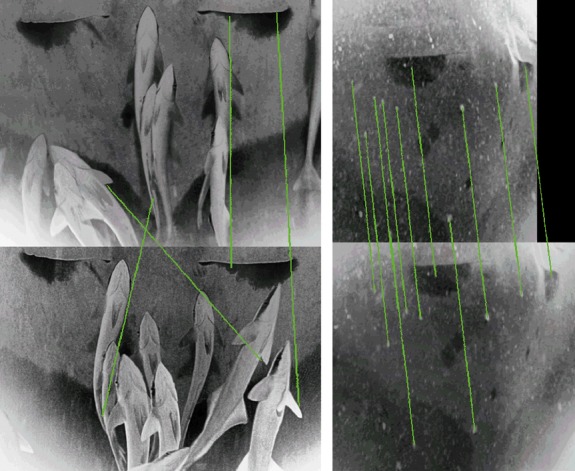
Examples of challenging matches where some features are matched incorrectly. Matched pairs of features are indicated by green lines drawn between them. Lack of visible high-contrast spot patterns, presence of occlusions, and extreme image noise can result in incorrect feature matches.

**Figure 9:**
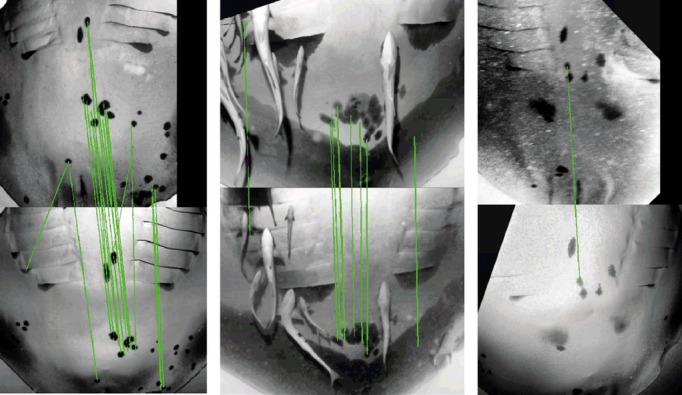
Examples of correct matches. Matching is robust to changes in viewing conditions and presence of occlusions.

We have also qualitatively analyzed the matching behavior of our algorithm in especially challenging cases. Figure 8 shows some examples where some of the SIFT features were matched incorrectly, but the manta rays were still identified correctly. On the other hand, as shows in Figure 9, our algorithm successfully copes with a wide variation in viewing conditions, poses, lighting, and noise. Images can in some cases be correctly matched on the basis of a very small subset of their features.

## Discussion

### Utility of automated visual identification

As noted in Marshall and Pierce (2012) and Speed et al. (2007), photographic identification has become an important tool in the study of population ecology; to assess biological variables such as size at maturity, gestation periods and reproductive periodicity, survivorship, growth, and longevity; and to investigate aspects of behavioral ecology including predation, social interactions, group feeding, visits to cleaning stations, and mating behavior.

Unlike human observers, automated matching techniques are consistent, free from subjective bias, and unaffected by fatigue. They allow researcher to harness much larger data sets than can feasibly be processed manually by enabling rapid automated identification of images with far less user effort. This is especially important for collaborative efforts that seek to bring together data sets from different research groups across the world: individual researchers may be sufficiently familiar with their own data, but large-scale global ecological data sets and the desire to incorporate sightings submitted by nonexperts clearly necessitate automated identification.

### Collaborative research on population ecology

The *MM* algorithm is being used as an important tool for a new global ecological database of manta ray sightings. Inspired by the success of the ECOCEAN^4^ Whale Shark Photo-identification Library, we have created a website^5^ which will serve as a global repository and collaborative tool for manta ray sightings. We are actively collating manta ray images from Mozambique, South Africa, Australia, Brazil, Ecuador, Thailand, Myanmar, Indonesia, Palau, Japan, Mexico, Hawaii, the Maldives, Peru, and New Zealand.

The website will serve as an important aid for research into manta ray ecology and biology, and we believe that the automated feature identification and matching methods described in this study will significantly contribute to its utility and efficacy. Moreover, increased automation will facilitate upload of photographic encounters by “citizen scientists”, thereby improving the scope and granularity of global sightings data and enhancing awareness for conservation efforts among the wider public.

## Summary

In this study we have described a new technique for automated identification of manta rays. Our method enhances images to remove noise and improve contrast to ameliorate the many problems affecting underwater photography. The characteristic markings on the ventral surface area of manta rays are then encoded through parameterized features, and a novel image-to-image comparison algorithm is capable of matching different images of the same ray. The method is robust to changes in viewpoint, scale, lighting conditions, pose, and occlusions. Unlike many other automated matching techniques, our approach requires only minimal user effort. In particular, users do not need to manually identify reference points on the images or perform elaborate image filtering in order to achieve good results. We have shown that our *MM* method correctly identifies over 84% of manta rays within the top 10% of ranked results in a data set of 720 manta ray images, with the majority of rays being correctly identified in the top-ranked image.

Our method has been deployed as the matching algorithm underlying a new global collaborative ecological database of manta ray sightings. In doing so we have demonstrated that modern pattern recognition techniques are powerful tools for ecological research. The ability to track individual animals can provide fine-grained information for fisheries management, and visually assessable parameters such as anthropogenic scarring provide vital information for ecological impact assessments and action planning.

Our research demonstrates that even species such as manta rays, whose characteristic markings are often indistinct and show substantial variability, can successfully be matched using automated photographic identification techniques, provided that the methods are sophisticated enough to deal with the highly diffuse nature of the spot patterns and the challenges of underwater imagery.

## Future Work

Our ongoing research seeks to further refine our *MM* method and investigate its suitability for other species. We are working on using automated segmentation algorithms to eliminate any need for manual image preprocessing, and we are also investigating the use of ancillary identifying information such as sex, maturity, color morphism (melanism or leucism), or scars.

In order to facilitate greater involvement by “citizen scientists”, we are planning to enable submission of manta ray sightings by amateur divers, and we are working on using our feature extraction techniques to define automated image fidelity assessments to maintain data quality and further improve the resilience of our *MM* algorithms in light of the large numbers of images we aim to process in future.

## Footnotes

1 http://www.reijns.com/i3s.

2 It relies on human judgment in distinguishing pigment marks and artifacts such as reflections, shadows, and particles in the water.

3 http://opencv.willowgarage.com/wiki/.

4 http://www.whaleshark.org.

5 http://www.mantamatcher.org.
